# Elderberry and Elderflower Extracts, Phenolic Compounds, and Metabolites and Their Effect on Complement, RAW 264.7 Macrophages and Dendritic Cells

**DOI:** 10.3390/ijms18030584

**Published:** 2017-03-08

**Authors:** Giang Thanh Thi Ho, Helle Wangensteen, Hilde Barsett

**Affiliations:** School of Pharmacy, Department of Pharmaceutical Chemistry, University of Oslo, P.O. Box 1068, Blindern, N-0316 Oslo, Norway; helle.wangensteen@farmasi.uio.no (H.W.); hilde.barsett@farmasi.uio.no (H.B.)

**Keywords:** *Sambucus nigra*, elderberry, elderflower, complement system, nitric oxide, anti-inflammatory, metabolites, polyphenols, LPS, RAW 264.7 cells, dendritic D2SC/I cells

## Abstract

Modulation of complement activity and inhibition of nitric oxide (NO) production by macrophages and dendritic cells may have therapeutic value in inflammatory diseases. Elderberry and elderflower extracts, constituents, and metabolites were investigated for their effects on the complement system, and on NO production in lipopolysaccharide (LPS)-activated RAW 264.7 macrophages and murine dendritic D2SC/I cells. The EtOH crude extracts from elderberry and elderflower and the isolated anthocyanins and procyanidins possessed strong complement fixating activity and strong inhibitory activity on NO production in RAW cells and dendritic cells. Phenolic compounds in the range of 0.1–100 µM showed a dose-dependent inhibition of NO production, with quercetin, rutin, and kaempferol as the most potent ones. Among the metabolites, caffeic acid and 3,4-dihydroxyphenylacetic acid showed the strongest inhibitory effects on NO production in both cell lines, without having cytotoxic effect. Only 4-methylcatechol was cytotoxic at the highest tested concentration (100 µM). Elderberry and elderflower constituents may possess inflammatory modulating activity, which increases their nutritional value.

## 1. Introduction

Inflammation is one of the organism’s attempts at self-protection, with the aim to remove harmful stimuli, including damaged cells, irritants, or pathogens. There is a growing research interest concerning anti-inflammatory natural products that will prevent and clarify the pathogenesis of various diseases such as cardiovascular diseases, diabetes, and chronic inflammatory diseases [1]. Numerous epidemiological studies indicate that an increase in the consumption of polyphenol-rich food is associated with a decrease in the incidence of cardiovascular diseases [1,2,3,4]. A large number of dietary polyphenols is consumed, and their anti-inflammatory activities have been reported [5]. This protective effect has been attributed in part to anti-inflammatory properties of polyphenols. At the level of bioactive compounds occurring in plants, substances such as polyphenols, polysaccharides, peptides, proteins, triterpenoids, lipid derivatives, and glycoproteins seem to modulate inflammatory as well as immunological processes [6,7].

The complement system plays an important role in the first-line defense against infections and holds important effector functions of the innate and the adaptive immune system. Complement participates in diseases with an immune component, such as autoimmune heart disease, multiple sclerosis, Alzheimer’s disease, rheumatoid arthritis, and severe bacterial infections [8,9]. The inflammation process involves the activation of monocytes and/or macrophages. The activation of macrophages releases many inflammatory mediators such as interleukins (IL-1β, IL-6), tumor necrosis factor (TNF)-α, and inflammatory mediators including reactive oxygen species (ROS), nitric oxide (NO), and prostaglandin E_2_ (PGE_2_). NO formed by inducible NO synthase (iNOS) plays a vital role in host defense as it possesses cytotoxic effects on bacteria, virus, and tumor cells [10]. On the other hand, in high concentrations it can lead to tissue damage and inflammatory diseases such as rheumatoid arthritis, cardiovascular diseases, type 2 diabetes, chronic hepatitis, and pulmonary fibrosis [11]. For this reason, NO is a well-established marker of inflammation, and inhibition of its production might be a useful therapeutic strategy in inflammatory diseases [10].

*Sambucus nigra* L., also called black elder, is a deciduous tree that can grow up to 10 m high and has blue-black elderberries and cream-white elderflowers [12]. Both the elderberries and elderflowers have a long tradition in herbal medicine of being used to reduce inflammation and diabetic symptoms, as diuretics, and in the treatment of colds and flu. Studies have shown that elderberry and elderflower extracts possess diverse biological activities such as anti-inflammatory, antioxidant, and antidiabetes effects [12,13]. *S. nigra* has components with high biological activity, primarily flavonoids, such as flavonols, proanthocyanidins and anthocyanins, and simpler phenolic acids [12]. However, due to the complex bioavailability of polyphenols, it is difficult to know what the most relevant substance is after intake of elderberries and elderflowers in humans. Due to the fact that polyphenols are metabolized to simpler phenolic substances [14], it was also of interest to investigate whether these metabolites could contribute to an effect. In human intervention studies involving feeding elderberry extracts and anthocyanins, many potential metabolites have been identified in urine, serum, and feces [15,16], and selected metabolites were included in this study.

In the present study, we systematically investigated the effects of the elderberry and elderflower extracts, constituents, and metabolites on the complement fixating activity and on the NO production in lipopolysaccharide (LPS)-stimulated RAW 264.7 macrophages and murine dendritic D2SC/I cells.

## 2. Results and Discussion

### 2.1. Extraction, Fractionation, and Chemical Characterization

**Figure 1:**
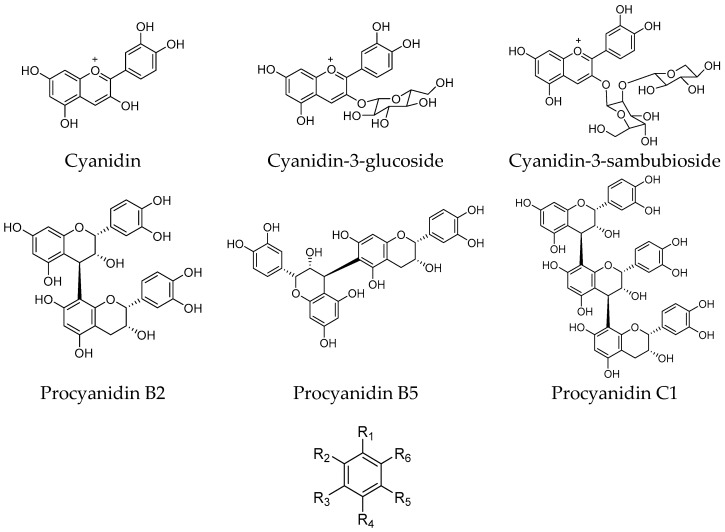
Structures of elderberry constituents and their metabolites.

**Figure 2:**
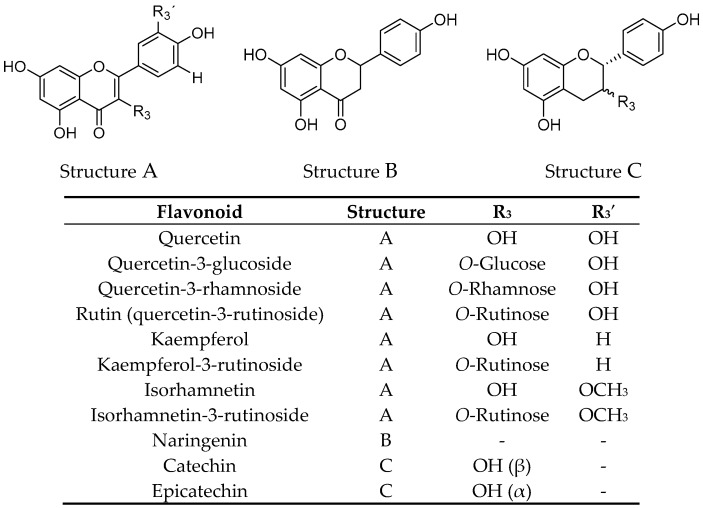

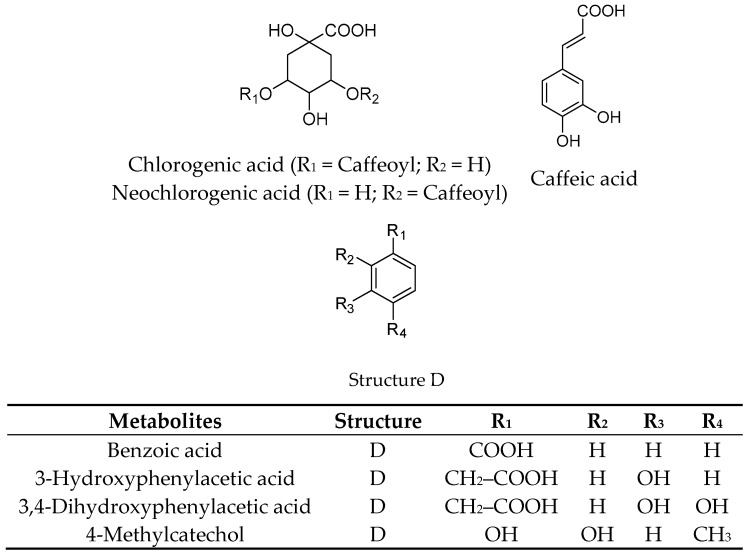
Structures of elderflower constituents and their metabolites.

Freeze-dried elderberries and elderflowers were extracted with dichloromethane (DCM), 96% EtOH, 50% EtOH, and water (50 and 100 °C) on an accelerated solvent extraction instrument (ASE). The phenol–sulfuric acid test [17] and ^1^H NMR analyses revealed that carbohydrates were present in the 50% EtOH and 50 and 100 °C water extracts. ^1^H and ^13^C NMR analyses of the EtOH elderberry and elderflower extracts showed signals from aromatic compounds, organic acids, and carbohydrates. The 96% EtOH elderflower extract showed signals which could be attributed to rutin [18] and caffeoyl moieties [19], structures previously reported to be major constituents in the elderflowers [20]. From the acidic elderberry MeOH extract (800 g), cyanidin-3-glucoside (340 mg, 0.043% yield) and cyanidin-3-sambubioside (250 mg, 0.031% yield), the major anthocyanins in elderberry, were isolated, in addition to the aglycone cyanidin (270 mg, 0.034% yield). The substances were identified by one- and two-dimensional NMR spectroscopy (^1^H, ^13^C, COSY, APT, HSQC, and HMBC) and the spectroscopic data were compared with those reported in the literature [21,22]. Chemical structures of the phenolic constituents and their metabolites from elderberries and elderflowers employed in this study are shown in Figure 1 and Figure 2.

### 2.2. Complement Fixating Activity

**Figure 3:**
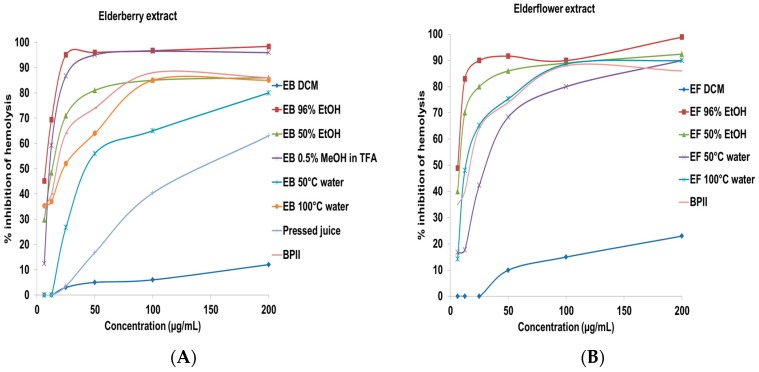

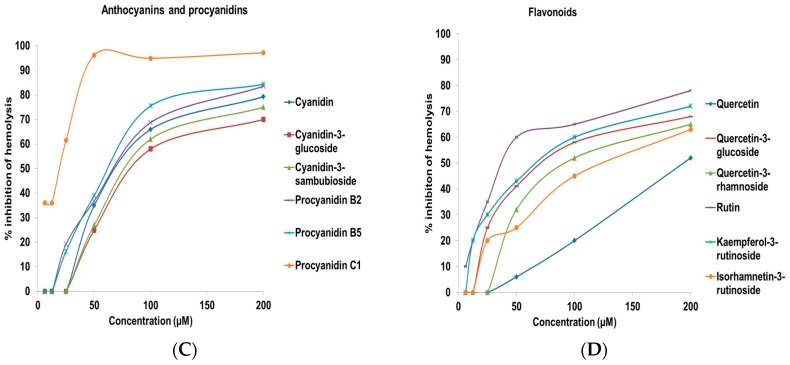
Complement fixating activity, expressed as % inhibition of hemolysis of sensitized sheep erythrocytes. Inhibition of hemolysis by (**A**) crude extracts from elderberry; (**B**) crude extracts from elderflower; (**C**) isolated anthocyanins and procyanidins; and (**D**) flavonoids. The results represent the mean of three independent experiments.

Due to the important role of the complement system in the immune system, complement modulation is involved in various diseases and considered as an interesting target for inflammatory diseases [23]. Elderberry and elderflower crude extracts, constituents, and metabolites were tested for complement fixating activity (Figure 3). The inhibitory concentration to give 50% effect (IC_50_) values are presented in Table 1 for the crude extracts and in Table 2 for the constituents and metabolites. A pectic polysaccharide from *Biophytum umbraculum* Welw. (syn. *B. petersianum* Klotzsch) was used as positive control [24,25].

Among the elderberry crude extracts, the acidic MeOH (IC_50_ 12.3 ± 1.9 µg/mL), the 96% EtOH (IC_50_ 7.8 ± 2.3 µg/mL), and the 50% EtOH (IC_50_ 13.4 ± 2.9 µg/mL) extracts showed the highest complement fixating activity. Thus, the acidic MeOH extract and the EtOH extracts have the highest selectivity for both polyphenols and components with high complement fixating activity. The 50 °C water and 100 °C water extracts containing polysaccharides also possessed complement fixating activities, in accordance with a previous report [26]. The IC_50_ values of cyanidin-3-glucoside and cyanidin-3-sambubioside, on a weight basis, are 18.0 µg/mL and 13.4 µg/mL, which is higher compared to the acidic elderberry MeOH extract (IC_50_ 12.3 µg/mL). There might be other strong complement fixating compounds in the acidic extract, or the effect is caused by synergism. The aglycone cyanidin showed somewhat higher activity compared to cyanidin-3-glucoside and cyanidin-3-sambubioside. The anthocyanins seem to have a lower inhibition of hemolysis compared to the corresponding aglycone. Proanthocyanidins that are present in small amounts in elderberries also possessed strong inhibitory activity on the hemolysis. Trimeric procyanidin C1 (IC_50_ 19.4 ± 2.1 µM) possessed stronger complement fixating activity compared to the dimeric procyanidins B2 (IC_50_ 70.6 ± 4.5 µM) and B5 (IC_50_ 65.0 ± 3.1 µM), while the monomers epicatechin and catechin were almost inactive (>200 µM). These results are in agreement with those reported previously [27,28]. Due to the fact that anthocyanins are metabolized to simpler phenolic substances, it was also of interest to investigate whether these metabolites that are detected in urine, feces and plasma after consumption of elderberries could have complement fixating activity. However, the metabolites shown in Figure 1 did not show any particular complement fixating activity from 0.1 to 200 µM.

The 96% EtOH and 50% EtOH elderflower extracts were the most potent inhibitors of hemolysis in the complement assay, followed by the water extracts. Pectic polysaccharides present in the water extracts might be a reason for the high complement fixating activity observed, in accordance with previous results [26,29,30]. Rutin (IC_50_ 40.0 ± 2.6 µM), quercetin-3-rhamnoside (IC_50_ 95.1 ± 3.1 µM), and quercetin-3-glucoside (IC_50_ 76.5 ± 2.6 µM) showed a higher complement fixating activity compared to the aglycone quercetin (IC_50_ 193.8 ± 5.6 µM), which is also reported previously [27,31]. Quercetin has even been reported to be inactive in a study [32]. The same trend is observed for kaempferol-3-rutinoside (IC_50_ 70.6 ± 3.8 µM) and its aglycone kaempferol (>200 µM) and for isorhamnetin-3-rutinoside (IC_50_ 127.8 ± 4.8 µM) and its aglycone isorhamnetin (IC_50_ > 200 µM). An earlier study showed that kaempferol and quercetin-3-rhamnoside had weak complement fixating activity with IC_50_ values 730 and 440 µM, respectively [32,33]. It appeared that the flavonol glycosides were more active than their aglycones in this system. These results are opposite to the activity of anthocyanins versus their aglycone. The presence of sugars seems to be important for complement fixating activity of the flavonoids included in this study. The metabolites from the elderflower phenolics (Figure 2) showed no particular complement fixating activity at the highest concentration tested (IC_50_ > 200 µM).

### 2.3. Inhibition of NO Production in LPS-Stimulated RAW 264.7 Macrophages and Dendritic D2SC/I Cells, and Cell Viability

**Figure 4:**
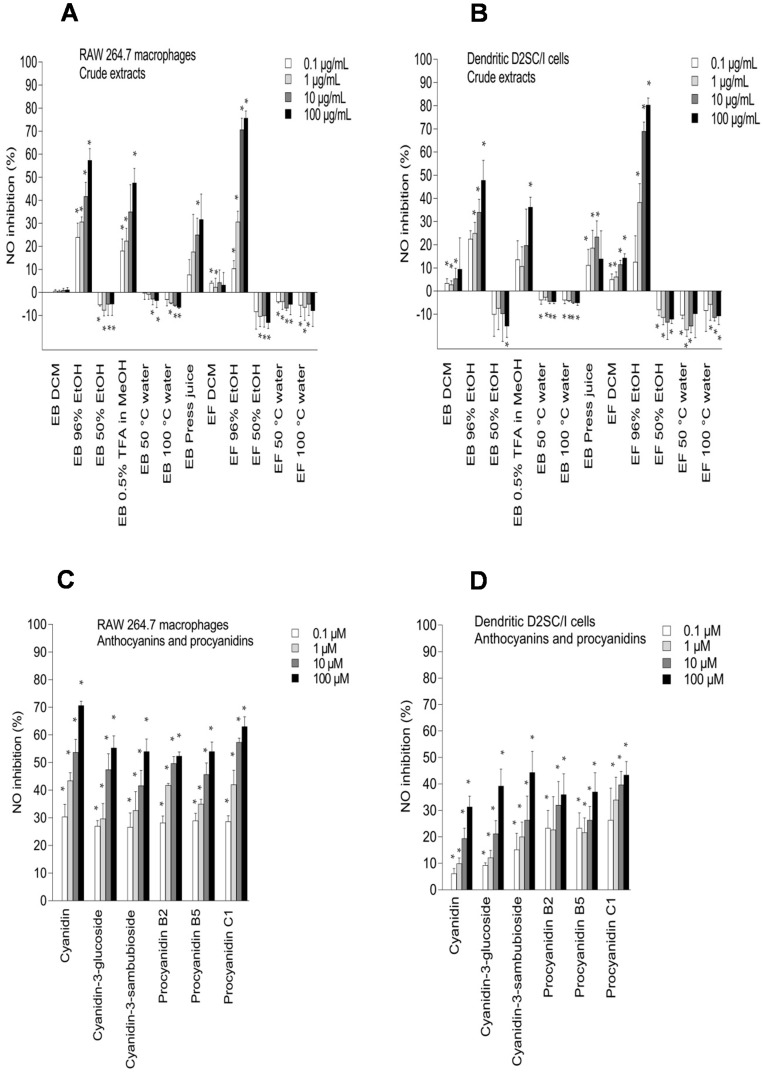

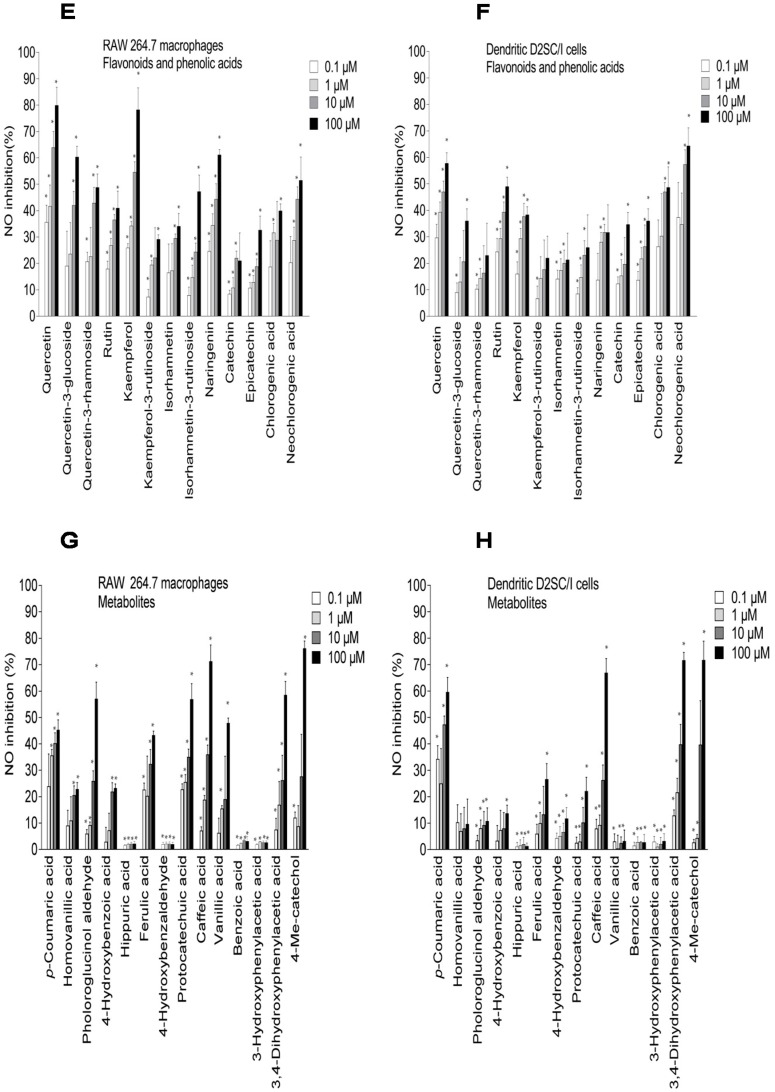
Inhibitory effects of elderberry (EB) and elderflower (EF) crude extracts (**A**,**B**); anthocyanins and proanthocyanidins (**C**,**D**); flavonoids and phenolic acids (**E**,**F**); and metabolites (**G**,**H**) on NO production in lipopolysaccharide (LPS)-activated RAW 264.7 macrophages (**A**,**C**,**E**,**G**) and dendritic D2SC/I cells (**B**,**D**,**F**,**H**). The experiments were repeated independently three times, and results shown are expressed as the average ± SEM. * *p* < 0.05 as compared to response of lipopolysaccharide (LPS) (0.1% DMSO) alone.

During inflammation, bacterial products and proinflammatory cytokines induce the formation of large amounts of nitric oxide (NO) by inducible nitric oxide synthase (iNOS), and compounds that inhibit NO production can be inflammatory modulators. Exposure of lipopolysaccharides (LPS) to macrophages triggers several disadvantageous cellular responses and causes responses similar to inflammation, sepsis, and stroke. Macrophages and dendritic cells play a major role in the initiation and modulation of immune responses [34]. An induction of iNOS in dendritic cells or macrophages via stimulation with LPS has been described in the literature [35]. The dendritic cells may function as the most potent antigen-presenting cells for T cell activation, and increased levels of NO may inhibit T cell proliferation and apoptosis in the dendritic cells [34]. The proposed mechanism associated with the inhibition of NO production in macrophages is scavenging of NO radicals, direct inhibition of iNOS enzyme activity, and/or inhibition of iNOS gene expression [11]. The inhibitory effect of elderberry and elderflower extracts, constituents, and metabolites on NO production in RAW 264.7 cells and dendritic D2SC/I cells exposed to LPS are shown in Figure 4. The NO concentration after exposure to the LPS control was 10.3 ± 2.1 µM for the RAW 264.7 cells and 14.6 ± 2.4 µM for the dendritic D2SC/I cells. Quercetin, which is a constituent in elderflower, was used as a positive control as well [11].

Among the crude extracts, the 96% EtOH elderflower extract (100 µg/mL) showed the highest NO inhibition in RAW 264.7 and dendritic cells (75.6% ± 3.1% and 80.3% ± 2.9%, respectively) followed by the 96% EtOH elderberry extract (57.3% ± 5.1% and 47.8% ± 8.7%, respectively) (Figure 4A,B). A high content of phenolic compounds, revealed by ^1^H NMR spectroscopy, in the 96% EtOH elderberry and elderflower extracts could be the reason for the high NO inhibitory activity. The DCM elderberry and elderflower extracts exhibited little effect on NO production in LPS-activated RAW 264.7 and dendritic D2SC/I cells. The water extracts induced the production of NO, explained by the presence of pectic polysaccharides, which previously have shown macrophage stimulation [26,29,30]. The elderberry and elderflower crude extracts did not show any cytotoxic effects against RAW 264.7 macrophages and dendritic D2SC/I cells at 100 µg/mL (81.2%–105.6% cell viability). Also, cold-pressed raw juice possessed moderate NO inhibition on LPS-activated RAW 264.7 and dendritic cells.

**Figure 5:**
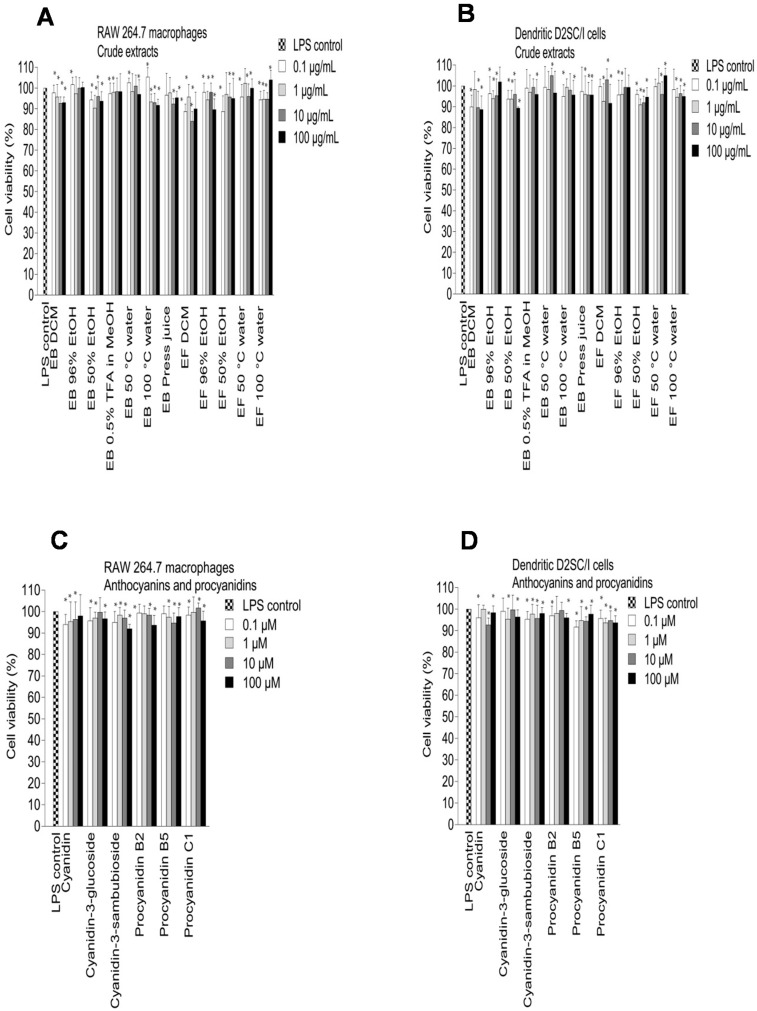

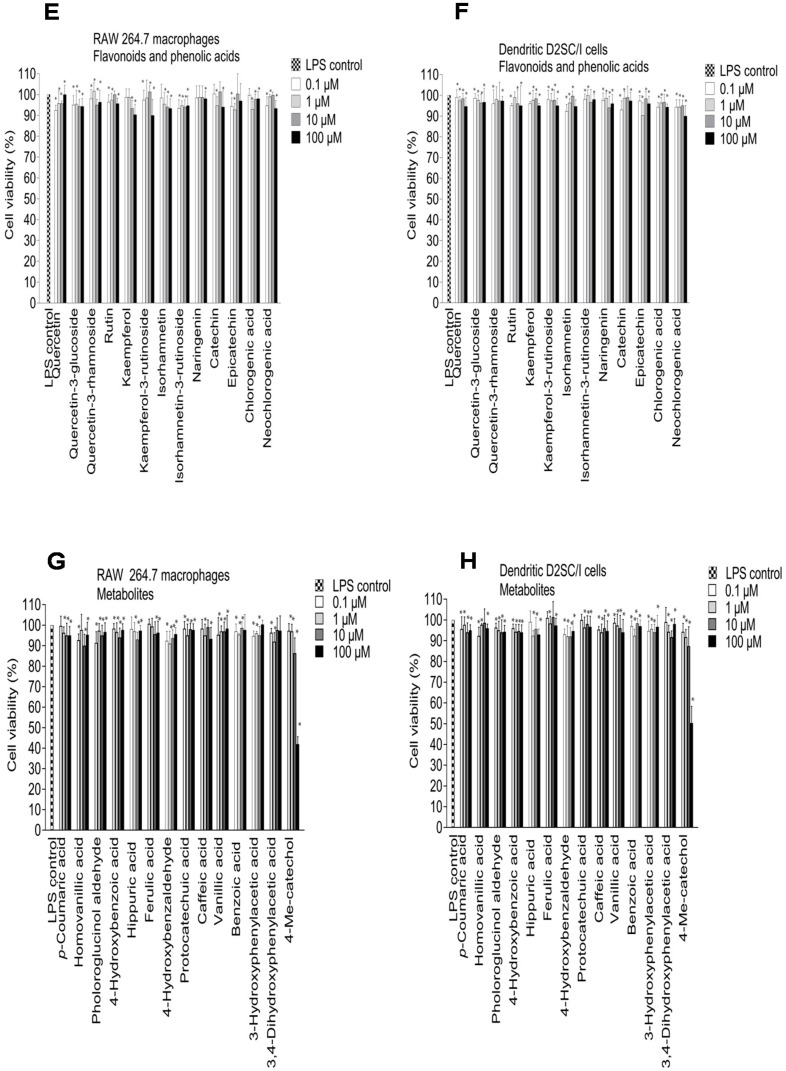
Effect of elderberry (EB) and elderflower (EF) crude extracts (**A**,**B**); anthocyanins and proanthocyanidins (**C**,**D**); flavonoids and phenolic acids (**E**,**F**) on cell viability in LPS-activated RAW 264.7 cells (**A**,**C**,**E**,**G**) and dendritic D2SC/I cells (**B**,**D**,**F**,**H**). Cell viability was assessed using the MTT assay. The experiments were repeated independently three times, and results shown are expressed as the average ± SEM. * *p* < 0.05 vs. LPS (0.1% DMSO) as control.

The elderberry anthocyanins and procyanidins showed high inhibitory effects on NO production (Figure 4C,D) and no toxicity towards the RAW 264.7 and dendritic D2SC/I cells (Figure 5C,D). The aglycone cyanidin showed a stronger inhibitory effect when compared to cyanidin-3-glucoside and cyanidin-3-sambubioside in the RAW 264.7 cells, and the effect shown was dose-dependent (Figure 4C). Cyanidin-3-glucoside and cyanidin-3-sambubioside, however, showed a higher inhibitory effect on NO production compared to its aglycone cyanidin in the dendritic cells at 100 µM (Figure 4D). The sugar moiety attached to cyanidin aglycone seems in any case to influence the NO inhibitory effect in both the RAW 264.7 cells and the dendritic D2SC/I cells. Cyanidin glycosides are present in high amounts in elderberries and may therefore be the most important contributor to the NO inhibition in the RAW 264.7 cells and the dendritic D2SC/I cells. The trimeric procyanidin C1 showed somewhat higher inhibitory activity compared to the dimers procyanidin B2 and procyanidin B5.

Quercetin-3-glucoside, quercetin-3-rhamnoside, and rutin (quercetin-3-rutinoside) showed NO inhibition of 60.4% ± 4.0%, 48.8% ± 5.1%, and 40.9% ± 6.5% at 100 µM in RAW 264.7 cells, respectively (Figure 4E), while the aglycone quercetin had a NO inhibition of 79.9% ± 6.9% at 100 µM. Glycosylated forms seemed to have a weaker NO inhibition in the RAW 264.cells. The same trend was also found for kaempferol and kaempferol-3-rutinoside. Glycosylation of the flavonoids might lead to increased hydrophilicity and steric hindrance, which again results in reduced absorption or penetration into the cells. This might influence the NO inhibitory effect in the RAW 264.7 cells. Similar results have also been reported previously [2,11]. NO inhibition by flavonoids in dendritic D2SC/I cells (Figure 4F) showed almost the same pattern as in RAW 264.7 cells, but generally the activity in dendritic cells was somewhat lower. Kaempferol showed a strong NO inhibition in the RAW 264.7 cells and weaker inhibition in the dendritic cells. Rutin (glycosylated with a disaccharide), however, was a more potent NO inhibitor in the dendritic cells than in the macrophages, and was also more active than the other quercetin-glycosides tested. Dendritic cells have many of the same properties as macrophages, but their surface phenotype and functional characteristics indicate that they belong to a unique lineage. The polyphenols might act on different cellular targets in the RAW 264.7 cells and dendritic cells. Epicatechin and catechin showed a small but significant inhibitory effect compared to the LPS control in both the RAW 264.7 cells, and showed similar inhibitory effect in dendritic D2SC/I cells. Chlorogenic acid has previously been reported to have no inhibitory effect in RAW 264.7 cells and in J774 macrophages [2,11]. In our study, chlorogenic acid showed NO inhibitory activity in the RAW 264.7 and dendritic D2SC/I cells, with the highest activity in dendritic cells. At the highest concentration tested (100 µM), neochlorogenic acid inhibited NO production with 64.3% ± 6.8% in the dendritic D2SC/I cells, and had somewhat lower activity 51.5% ± 8.8% in RAW cells. Both rutin and chlorogenic acid are present in high amounts in elderflower [20] and may therefore be the most important contributors to modulation of inflammatory effects.

The polyphenols are absorbed rather poorly from the gut and are extensively metabolized in vivo, suggesting that the accumulation of multiple phenolic metabolites may be responsible for some of the reported bioactivity of the polyphenols. The metabolites showed somewhat similar effects of NO inhibition in macrophages and dendritic cells. However, there were some variations in activity from one compound to the other. Phloroglucinol aldehyde, protocatechuic acid, and vanillic acid showed a considerably higher inhibitory effect on NO production in the RAW 264.7 cells compared to the dendritic cells. Caffeic acid, 3,4-dihydroxyphenylacetic acid, protocatechuic acid, and phloroglucinol aldehyde were the most active metabolites, which inhibited the NO production in LPS-activated RAW 264.7 cells by 71.3% ± 6.2%, 58.5% ± 5.1%, 57.1% ± 6.2%, and 56.9% ± 5.8%, respectively, at 100 µM. Protocatechuic acid, the major metabolite from cyanidin-3-glucoside, showed the same inhibitory effect on NO production compared to cyanidin 3-glucoside in RAW 264.7 cells at 100 µM (56.9% ± 5.9% and 55.3% ± 4.0%, respectively). Previous studies have reported that protocatechuic acid and caffeic acid have strong anti-inflammatory properties [36,37]. In LPS-activated dendritic D2SC/I cells, 3,4-dihydroxyphenylacetic acid, caffeic acid, and *p*-coumaric acid were the most potent metabolites that inhibited NO production by >50% at 100 µM without showing cytotoxicity. 4-Methylcatechol was toxic at 100 µM against RAW 264.7 cells and dendritic D2SC/I cells (45.3% ± 3.6% and 51.2% ± 8.1% cell viability, respectively) and the strong NO inhibition observed for 4-methylcatechol at 100 µM could be caused by cell cytotoxicity (Figure 5G,H).

The bioavailability of elderberry anthocyanins is reported to be low; less than 0.1% of the ingested dose has been detected in humans [38]. Concentrations of 0.1–0.4 µM have been detected for the elderberry anthocyanins in blood and urine after intake of elderberry extract (500–700 mg anthocyanins) [15,16]. The bioavailability of the anthocyanin metabolites was reported to be 60- and 45-fold higher than their parent compounds based on concentrations in urine and plasma, respectively [15,16]. Bioavailability studies in humans of elderflower and their phenolic compounds are still missing. However, Williamson and Manach [39] reported that polyphenol concentrations of less than 10 µM may have physiological effects. This indicates that concentrations used in this study might have a clinical relevance.

As far as we know, this is the first study comparing the inhibitory effects on NO production in LPS-activated RAW 264.7 cells and dendritic D2SC/I cells of elderberry and elderflower extracts. In addition, this study gives a comprehensive overview of complement fixating activity and NO inhibition in RAW 264.7 and dendritic cells of the major phenolic compounds present in elderberry and elderflower. Moreover, for the first time we showed that the metabolites in physiological concentrations inhibited the NO production in macrophages and dendritic cells. In sum, these results bring new and supportive data to the inflammatory modulating properties of the phenolic compounds present in elderberries and elderflowers. Thus, intake of elderberry and elderflower might help to regulate inflammatory diseases.

## 3. Materials and Methods

### 3.1. Plant Material

Fresh elderberries (cultivar “Sampo”) and raw elderberry juice (made by cold-pressing followed by filtering to remove peels and seeds and heating to 68 °C) were gifts from cultivator Rune Hatleli, Fresvik in Sogn, Norway. The berries were harvested in October 2014 and stored at −20 °C until extraction. Certified ecologically cultivated flowers of *Sambucus nigra* L., harvested in Norway October 2012, were purchased from Odins Marked, Oslo, Norway (org. No: 876905892) in November 2012. Voucher specimens of elderberries (No. EB1010) and elderflowers (No. EF1020) are deposited in the Pharmacognosy section, School of Pharmacy, University of Oslo, Norway.

### 3.2. Chemicals

4-Hydroxybenzaldehyde, 4-hydroxybenzoic acid, caffeic acid, homovanillic acid, vanillic acid, protocatechuic acid, phloroglucinol aldehyde, hippuric acid, ferulic acid, *p*-coumaric acid, quercetin, chlorogenic acid, neochlorogenic acid, epicatechin, catechin, kaempferol, kaempferol-3-rutinoside, 3-hydroxyphenylacetic acid, 3,4-dihydroxyphenylacetic acid, isorhamnetin-3-rutinoside, naringenin, isorhamnetin, rutin, quercetin, quercetin-3-rhamnoside, quercetin-3-glucoside, 4-methylcatechol, benzoic acid, DMSO-*d*_6_, CD_3_OD, Griess reagent A, Griess reagent B, cell proliferation kit I (MTT), and bovine serum albumin (BSA) were purchased from Sigma-Aldrich (St. Louis, MO, USA). Proanthocyanidins B2, B5, and C1 were obtained from Plantchem (Klepp, Norway). Dulbecco‘s modified Eagle’s medium (DMEM-Glutamax™, 5.5 mM), fetal bovine serum, and penicillin–streptomycin–amphotericin B were obtained from Gibco Life Technologies (Paisley, UK). Corning CellBIND tissue culture plates were obtained from Corning Life-Sciences (Schiphol-Rijk, The Netherlands). All other reagents were of the highest purity available.

### 3.3. Extraction and Isolation of Anthocyanins

Elderberries and elderflowers were extracted according to Ho et al. [40]. For extraction, 30 g freeze-dried berries or flowers were mixed with diatomaceous earth (Dionex, Sunnyvale, CA, USA) (4:1) and loaded in a 100 mL steel cartridge. The elderberries and elderflowers were then extracted successively with dichloromethane (DCM) (~900 mL for elderberries and ~700 mL for elderflowers), followed by 96% EtOH (~950 mL for elderberries and ~800 mL for elderflowers), 50% EtOH (~650 mL for elderberries and ~500 mL for elderflowers), 50 °C water (~450 mL for elderberries and ~300 mL for elderflowers), and 100 °C water (~450 mL for elderberries and ~300 mL for elderflowers). The anthocyanins from elderberries were isolated in accordance to Bräunlich et al. [41]. Briefly, freeze-dried elderberries were extracted by maceration with MeOH (0.5% trifluoroacetic acid (TFA) *v*/*v*) for 24 h. The dried extract was applied on an Amberlite XAD-7HP column (5 cm × 50 cm) with water followed by MeOH (0.5% TFA) as mobile phase. The anthocyanin-enriched fraction was then fractionated on a Sephadex LH-20 column (5 cm × 100 cm) (water–MeOH (0.5% TFA) as gradient), and purified by preparative HPLC using a Microsorb 60-8 C_18_ (250 mm × 21.4 mm) column (Varian, Palo Alto, CA, USA) with a water-acetonitrile (0.5% TFA) gradient as eluent.

### 3.4. NMR Spectroscopy

^1^H and ^13^C nuclear magnetic resonance (NMR) was conducted on a Bruker DPX 300 or a Bruker AVII 400 instrument (Bruker, Rheinstetten, Germany) with CD_3_OD, CD_3_OD:TFA (95:5) or DMSO-*d*_6_ as solvents and tetramethylsilane (TMS) as reference.

### 3.5. Complement Fixating Assay

The complement fixating test is based on inhibition of hemolysis of antibody-sensitized sheep red blood cells (SRBC) by human sera, as described by Michaelsen et al. [42] (Method A). Samples were dissolved in 0.1% DMSO or veronal buffered saline (VBS) pH 7.2 containing 0.2 mM CaCl_2_ and 0.8 mM MgCl_2_ with 2 mg/mL bovine serum albumin (BSA). A pectic polysaccharide, BPII, from *Biophytum umbraculum* Welw. (syn. *B. petersianum* Klotzsch) was used as a positive control [25]. A dose–response curve was constructed to calculate the concentration of the test sample that gave 50% inhibition (IC_50_) of hemolysis. The experiments were repeated independently three times.

### 3.6. RAW 264.7 and Dendritic D2SC/I Cells

The mouse macrophages cell line RAW 264.7 and dendritic cell line D2SC/I were cultured in Dulbecco’s modified eagle medium (DMEM) supplemented with 10% fetal bovine serum, 1% 100 mM sodium pyruvate, 1% Pen Strep (10,000 U/mL penicillin and 10,000 µg/mL streptomycin), and 5 × 10^−5^ M 2-mercaptoethanol and maintained in a 37 °C humidified incubator containing 5% CO_2_.

### 3.7. Nitrite Assay

The nitrite assay was carried out as previously described with minor modifications [28]. Macrophages and dendritic cells at a density of 5 × 10^5^ cells/mL were seeded into 96-well flat-bottomed plates and pre-incubated with the test samples for one hour before the addition of 500 ng/mL LPS (*Escherichia coli* O55:B5, Sigma-Aldrich). Samples were dissolved in DMSO (0.1% final concentration) or medium. The cells were incubated for 24 h in duplicates containing 0.1, 1, 10, and 100 µg/mL (extracts) or 0.1, 1, 10, and 100 µM (pure compounds) of test samples (final concentrations). NO was determined by measuring the amount of nitrite, which can be measured in a colorimetric assay using the Griess reagents. The supernatant (50 μL) was mixed with 50 μL of Griess reagent A (1% sulfanilamide in 5% phosphoric acid) and incubated at room temperature in the dark for 10 min. Then 50 μL Griess reagent B (0.1% *N*-(1-naphthyl) ethylenediamine dihydrochloride in water) was added, and the absorbance was measured at 540 nm using a Bio-Rad reader. Quercetin was used as a positive control. A serial dilution of NaNO_2_ in medium was used to construct the standard reference curve. Percentage NO inhibition was expressed as the percentage decrease in NO production as: 100 − [NO]^a^/[NO]^b^ × 100, where [NO]^a^ represents the NO concentration for test samples and [NO]^b^ represents the NO concentration from LPS-activated control. The experiments were repeated independently three times.

### 3.8. Cell Viability Assay

Cell viability was determined by the MTT assay. The assay is based on the cleavage of the yellow tetrazolium salt MTT to purple formazan crystals by metabolic active cells. The method was done according to the manufacturer’s procedure [43]. Briefly, RAW 264.7 and dendritic D2SC/I cells were seeded into 96-well plates at a density of 5 × 10^5^ cells/well and incubated with test samples (0.1, 1, 10, and 100 µg/mL (extracts) or 0.1, 1, 10, and 100 µM (pure compounds)) for 24 h in a humidified 6.5% CO_2_ atmosphere at 37 °C. After the incubation period, 10 µL of MTT reagent was added to each well. After 4 h, 100 µL of solubilization solution was added. The quantity of formazan (presumed to be directly proportional to the number of viable cells) is measured by recording changes in absorbance at 595 nm using a microplate spectrophotometer (iMark Microplate reader, Bio-Rad, ’Hercules, CA, USA). Medium was used as negative control and 20% DMSO as positive control. The experiments were repeated independently three times.

### 3.9. Statistical Analysis

Each assay was performed in duplicate or triplicate and results are given as mean (±SEM) from three separate experiments. Comparisons of different treatments were evaluated by two-tailed Student’s *t*-test. Statistical analyses were performed using GraphPad Prism 5.0 for Windows (GraphPad Software, San Diego, CA, USA).

## Figures and Tables

**Table:** 

Chemical Compound	R_1_	R_2_	R_3_	R_4_	R_5_	R_6_
*p*-Coumaric acid	CH=CH–COOH	H	H	OH	H	H
Homovanillic acid	CH_2_–COOH	H	H	OH	OCH_3_	H
Phloroglucinol aldehyde	CHO	OH	H	OH	H	OH
4-Hydroxybenzoic acid	COOH	H	H	OH	H	H
Hippuric acid	CO–NH–CH_2_–COOH	H	H	H	H	H
Ferulic acid	CH=CH–COOH	H	OCH_3_	OH	H	H
4-Hydroxybenzaldehyde	CHO	H	H	OH	H	H
Protocatechuic acid	COOH	H	H	OH	OH	H
Caffeic acid	CH=CH–COOH	H	H	OH	OH	H
Vanillic acid	COOH	H	H	OH	OCH_3_	H

**Table 1:** Complement fixating activity of crude extracts from elderberry (EB) and elderflower (EF). DCM, dichloromethane; TFA, trifluoroacetic acid.

Test Compounds	IC_50_ (µg/mL) ^a^
**Crude extracts from elderberry**	
EB DCM	>200
EB 96% EtOH	7.8 ± 2.3
EB 50% EtOH	13.4 ± 2.9
EB 0.5% TFA in MeOH	12.3 ± 1.9
EB 50 °C water	44.9 ± 5.3
EB 100 °C water	23.3 ± 3.5
Pressed juice	142.4 ± 13.1
**Crude extracts from elderflower**	
EF DCM	>200
EF 96% EtOH	6.5 ± 1.5
EF 50% EtOH	8.9 ± 2.2
EF 50 °C water	32.3 ± 5.2
EF 100 °C water	14.4 ± 3.1
BPII (positive control)	17.7 ± 1.5

^a^ IC_50_: Concentration to give 50% inhibition of hemolysis.

**Table 2:** Complement fixating activity of flavonoids and metabolites from elderberries and elderflowers.

Test Compounds	IC_50_ (µM) ^a^
**Flavonoids**	
Cyanidin	74.2 ± 3.4
Cyanidin-3-glucoside	87.1 ± 5.6
Cyanidin-3-sambubioside	82.8 ± 3.9
Procyanidin B2	70.6 ± 4.5
Procyanidin B5	65.0 ± 3.1
Procyanidin C1	19.4 ± 2.1
Quercetin	193.8 ± 5.6
Quercetin-3-glucoside	76.5 ± 2.6
Quercetin-3-rhamnoside	95.1 ± 3.1
Rutin	40.0 ± 2.6
Kaempferol	>200
Kaempferol-3-rutinoside	70.6 ± 3.8
Isorhamnetin	>200
Isorhamnetin-3-rutinoside	127.8 ± 4.8
Naringenin	>200
Catechin	>200
Epicatechin	>200
**Metabolites**	
*p*-Coumaric acid	>200
Homovanillic acid	>200
Phloroglucinol aldehyde	>200
4-Hydroxybenzoic acid	>200
Hippuric acid	>200
Ferulic acid	>200
4-Hydroxybenzaldehyde	>200
Protocatechuic acid	>200
Caffeic acid	>200
Vanillic acid	>200
Benzoic acid	>200
3-Hydroxybenzoic acid	>200
3,4-Dihydroxyphenylacetic acid	>200
4-Methylcatechol	>200

^a^ IC_50_: Concentration to give 50% inhibition of hemolysis.

## Abbreviations

DCM	Dichloromethane
DMSO	Dimethylsulfoxide
EB	Elderberry
EF	Elderflower
EtOH IC_50_	Ethanol Inhibitory concentration to give 50% effect
LPS	Lipopolysaccharide
MeOH	Methanol
MTT	3-(4,5-dimethylthiazol-2-yl)-2,5-diphenyltetrazolium bromide
NO	Nitric oxide
NMR	Nuclear magnetic resonance
TMS	Tetramethylsilane
TFA	Trifluoroacetic acid
